# The Treatment of Nearly Exposed Pulps

**Published:** 1893-07

**Authors:** C. F. Ives

**Affiliations:** New York


### THE TREATMENT OF NEARLY EXPOSED PULPS.[1]

BY DR. C. F. IVES, NEW YORK.

WHEN asking me to give a brief "communication" on the treatment of "nearly exposed pulps," the chairman of the Executive Committee said, "It is a long time since we have had a talk on 'pulps,' and it will do us all good to stir up a discussion." I have really nothing new to say, nothing original to offer: an experience or two, some confessions of failures which set me to thinking; and you all know that when we sit down to the task of honestly criticising our own failures some good results are apt to be evolved. I will simply tell you how I treat these cases. If it prove to be "ancient history," it will at least provoke discussion, and so we may gain a point.

By nearly exposed pulps I do not mean those cases where, if you lift the last layer of semi-soft decay, a living pulp presents itself, but those where a fair amount of sound dentine intervenes, and you are in dangerous proximity. If you recognize the situation, proceed to place some non-conducting substance on the floor of the cavity before introducing a metallic filling, and you will find, after one, two, or more years, a dead pulp, and you wonder why! If, on carefully removing the filling, no non-conductor is found, but sound bone, and the filling too near the pulp, its death was the result of thermal changes.

My first failure was a case of my own. A large approximal cavity in a superior central, no exposure, a capping of oxyphosphate preceded by two or three coats of varnish, then a filling of gold.

---

[1] Read before the New York Odontological Society, March 21, 1893.

Five years of quiet, then pain. A careful removal of gold and cement revealed no decay, only as the burr entered the chamber the layer of dentine was a little thinner than I had anticipated, but it was thoroughly protected. It was not the oxyphosphate, or thermal changes, but pressure.

That same year I had an exceptionally large number of cases not my own work. Being greatly interested, I was careful to remove the fillings, first placing on the dam. In many I found a fair layer of sound dentine over the dead pulp. In one—a large crown approximal cavity in a superior molar—the operator had more than half filled it with gutta-percha, over which was amalgam. It was very evident what had killed that pulp. In many others metallic fillings had been placed directly on the floors of the cavities.

I think that in comparatively painless excavations, with the feeling of sound bone under the burr or excavator, we are very apt to take chances perhaps not warranted, forgetting that one thing a pulp will not submit to is *pressure*, and we are often a trifle nearer the dividing line than we are aware of. Here is my point. You cannot place directly over a nearly exposed pulp as a non-conductor either gutta-percha in bulk or solution, oxyphosphate, oxide of zinc with carbolized gum-water, or any compound that will harden, without producing pressure from expansion or contraction. It may be slight, but, slight as it is, I know it often results in the death of the pulp. Gutta-percha, tissue-cork, quill, or whatever may be your favorite capping, if lying directly on the floor of the cavity and covered with other material which requires even light packing, will often prove fatal to the nearly exposed pulp.

I endeavor to avoid all pressure. A piece of very thin aluminum,—this because of its purity and lightness,—a bit of hard wood, apple-seed, or shot-head burnishers, and in a moment a concave cap is prepared to fit the case (this dipped in varnish); the cavity bathed with oil of cinnamon (not oil of cassia, which is the Chinese product, but the Ceylon oil, which you will know, because it will cost you two dollars an ounce), for which I have discarded all other antiseptics in cavities, because it is safe, effective, and pleasant. The cap, always large enough to rest outside the line of the chamber, is covered with a layer of "Fletcher's Nerve-Capping." If you do not use it, let me urge you to try it. For thirteen years it has proved in my hands an efficient aid in all such cases. It is an oxysulphate of zinc, non-escharotic, and hardens sufficiently in three or four minutes to pack any material upon. It is a beautiful temporary filling, wonderfully nice for cavity lining, and, in combination with any good antiseptic, an excellent root-filling. I always cover my application of arsenical paste with this cap and Fletcher's material, avoiding all pressure. Dr. Miller, of Berlin, has recently spoken very enthusiastically of it.

Let me tell you of an excellent varnish. Procure a piece of clear amber, scrape or powder it, dissolve in Squibb's chloroform, which will take some time, add a little absolute alcohol to delay evaporation, and you have a varnish so hard that it will resist almost anything.

As this is a family talk, let me say a word or two about exposed pulps. I confess right here that, to the best of my knowledge, I never saved one in my life, and I have faithfully and honestly tried. I have nothing to say against your trying, for, to paraphrase some one else, "I need not compound for sins I am inclined to by damning those I have no mind to," but it is positively wicked for me to attempt this any more. If ever the time comes, however, when the operation is an assured success, one great factor will be absolute freedom from pressure. I have had some disagreeable cases to treat which I consider the result of destroying pulps by driving a splint of wood up as far as possible,—"pulp-condensing." If any of you use this method, inform us, when your turn comes, why!

Now I have said just enough to start the subject, and from your various experiences we shall all have something to take home with us for edification.

